# *Lactobacillus acidophilus* novel strain, MJCD175, as a potential probiotic for oral health in dogs

**DOI:** 10.3389/fvets.2022.946890

**Published:** 2022-09-02

**Authors:** Inhwan You, Feriel Yasmine Mahiddine, Heekee Park, Min Jung Kim

**Affiliations:** Department of Research and Development, Mjbiogen Corp., Seoul, South Korea

**Keywords:** probiotic, oral health, periodontal disease, dental caries, canine (dog)

## Abstract

The oral cavity is the second-largest habitat for microorganisms, and a well-balanced oral microbiome contributes to preventing dental disorders caused by pathogenic bacteria. Since humans and dogs have different lifestyles and oral microbiome structures, the present study aimed to develop novel probiotics for dogs. A total 53 *Lactobacillus* spp. were isolated from healthy dogs, and nine isolates were identified as *Lactobacillus acidophilus* according to 16S rRNA gene sequencing. According to the high antimicrobial activity against the dental caries-causing bacterium *Streptococcus mutans*, single or three mixed strains were orally administered to dogs for 4 weeks with concentration of 10^8^-10^9^ CFU/day. Intraoral swab samples were collected before and after the administration, and changes of oral pathogen were analyzed using quantitative PCR. Among them, *Porphyromonas gingivalis*, a critical factor of periodontitis, was significantly reduced in the single-strain administered group. Based on the acid and bile salts tolerance characteristics of isolates, systemic effects were also analyzed by comparing serum immunoglobulin and reproductive ability before and after the administration. However, no significant changes were observed in the serum IgG level and sperm quality. Overall, these *in vitro* and *in vivo* results suggest that *L. acidophilus* isolates from dogs, especially *L. acidophilus* MJCD175, could be promising probiotic candidates to support oral health without systemic adverse effects in dogs.

## Introduction

Dental disorders caused by bacteria, such as dental caries and periodontal disease, are the most common oral problems in small animals. Dental caries (or tooth decay) is rare in dogs compared to other dental diseases, and 5.3% of adult dogs have one or more carious lesions (1). Tooth decay causes damage and tooth loss and is caused by the acid produced by the bacteria *S. mutans*, which is the main causative bacteria (1). Periodontal disease is one of the most common conditions in companion dogs, with over 80% prevalence (2), and its symptoms include infection and inflammation of the gums, bad breath, and loosing tooth. Although several factors, such as age, sex, living environment, and the size of the dog, are associated with the prevalence of periodontal disease in dogs (3), the leading cause of the periodontal disease is specific pathogenic bacteria (i.e., *Porphyromonas gingivalis*) residing in biofilms (4). Thus, it is an inflammatory condition between the host immune response and subgingival biofilm (5).

In humans, the oral cavity is the second-largest reservoir of bacteria after the gut, with over 700 species of microorganisms (6). Maintaining healthy oral microbiota is the most promising strategy to reduce the development of dental caries and prevent periodontitis. For example, a well-balanced oral microbiome can resist the constantly changing external factors, including colonization by foreign microbes and pathogens (7). Both symbionts and pathobionts are part of the oral microbiome, but pathobionts become dominant when the microbial balance is disrupted, leading to tooth decay and periodontitis (8–11). Besides, the oral microbiome is closely linked to other systemic diseases, including rheumatoid arthritis and intestinal inflammatory diseases (12–15). As the first gateway to the immune and digestive systems, a healthy and balanced oral microbiome is vital for oral and overall health.

Probiotics are beneficial microorganisms that promote host health *via* microbial management (16). During the past few decades, many studies have demonstrated that probiotics, especially *Lactobacillus* spp., manage the gut microbiome as well as the oral microbiome (17, 18). Probiotics contribute to maintaining and ameliorating oral health through direct or indirect competition with oral pathogenic bacteria (19–21). Additionally, the action of probiotics includes the replacement of opportunistic pathogens with beneficial bacteria, inhibition of attachment of pathogenic bacteria, combating bacterial biofilms, and anti-inflammatory activity (22–24).

*Lactobacillus acidophilus* is a critical probiotic and is accepted as a non-strain-specific probiotic core group by Health Canada and some European Union countries (25, 26). In addition to oral health supplements, live cells, dead cells, or metabolites of *L. acidophilus* are used in boundless fields such as dietary supplements, food, medicine, and cosmetics. However, most *in vivo* studies have been performed in humans using *L. acidophilus* strains isolated from humans or the environment. Thus, probiotic products for dogs are based on human *in vivo* results. However, dogs have different habits than humans, such as food intake, tooth brushing, licking, and a different saliva pH, and only 16.4% of oral taxa are shared between dogs and humans (27). This study evaluated the effect of novel *L. acidophilus* strains isolated from dogs on their oral health.

## Materials and methods

### Bacteria isolation and identification

Fresh fecal samples from 22 healthy dogs were collected and directly used for further experiments. Fecal samples were diluted with phosphate-buffered saline (PBS) and plated on de Man Rogosa and Sharp (MRS; MB cell, Seoul, Korea) agar medium. After overnight incubation at 37°C, a single colony was isolated. To identify the bacterial morphology, Gram staining was performed according to the manufacturer's recommendations (BD Difco, New Jersey, USA). To select *Lactobacillus* strains, only gram-positive, bacilli-shaped bacteria were selected.

For *Lactobacillus* sp. selection, a previously reported genus-specific primer pair and PCR condition were used (28): LbLMA1-rev, 5′-CTCAAAACTAAACAAAGTTTC-3′ and R16-1, 5′-CTTGTACACACCGCCCGTCA-3′. Strains showing a band size of ~250 bp on electrophoresis were assumed to be *Lactobacillus* spp.

Selected strains were identified as potential probiotics by 16S ribosomal RNA sequencing. Genomic DNA was extracted using a G-spin kit (Intron, Seoul, Korea) and amplified by polymerase chain reaction (PCR) using the universal primer pair targeting the V1–V9 region of 16S rRNA (27F, 5′-AGAGTTTGATCMTGGCTCAG-3′ and 1492R, 5′-TACGGYTACCTTGTTACGACTT-3′). The PCR protocol was comprised of initial denaturation at 95°C for 3 min followed by 30 cycles containing 95°C for 30 s, annealing at 50°C for 30 s and extension at 72°C for 1 min and final extension step at 72°C for 10 min. Then, purified PCR products were sequenced with the universal primer pair (785F, 5′-GGATTAGATACCCTGGTA−3′ and 907R, 5′-CCGTCAATTCMTTTRAGTTT-3′). The sequences resulting from Sanger sequencing were identified using the basic local alignment search tool (BLAST) based on sequence coverage and similarity.

### Antimicrobial activity

To develop strain-specific antimicrobial activity, four different pathogens were used (*Candida albicans* KACC 30071, *Escherichia coli* KACC 15541, *Staphylococcus aureus* KACC 13236, and *Streptococcus mutans* KACC 16833). The inhibitory activity of the supernatant of the isolates against pathogens was carried out based on Danilova et al. with some modifications (29). The isolates were cultured as mentioned above, and the supernatants were harvested from fresh cultures of the isolates by centrifugation at 13,000 rpm for 2 min. Pathogens were obtained from the Korea Agricultural Culture Collection (KACC). Obtained pathogens were cultured in TSB broth at 37°C for overnight. The resulting supernatants (20%, v/v) were added to the MRS broth inoculated with pathogenic bacteria at a concentration of 1% (v/v). MRS broth inoculated with pathogenic microorganisms without the supernatant was used as a control. The optical density (OD) values were measured at 600 nm for 18 and 24 h at 37°C each.

### Acid and bile salts tolerance

Acid and bile tolerance of three isolates which showed the strongest inhibition activity against *S. mutans* were assessed under gastrointestinal tract-like conditions. Overnight bacterial culture inoculated (1%, v/v) into MRS broth medium adjusted to pH 3.0 ± 0.05 with 1N HCl (Sigma-Aldrich, MO, USA) using an Orion Star™ A211 pH Benchtop Meter (Thermo Scientific, MA, USA). The number of bacterial colony-forming units (CFU) was determined on MRS agar plates at 0 and 2 h incubation at 37°C. For the suppression rate in bile salt, fresh bacterial culture prepared in the same conditions mentioned above were used. Bacterial cultures were inoculated (1%, v/v) into MRS broth medium and MRS broth medium containing 0.3% (w/v) oxgall (Sigma-Aldrich, MO, USA), respectively. OD_600_ was measured for 0 h and 12 h at 37°C.

### Antibiotic susceptibility profile

Antibiotic resistance profiles were elucidated using the disk diffusion assay (30). Fresh overnight bacterial cultures of each isolate were spread onto MRS agar plates, and antibiotic disks were placed on agar plates. A total of 13 antibiotic disks were used: rifampicin (RIF, 5 μg), ciprofloxacin (CIP, 5 μg), vancomycin (VAN, 30 μg), streptomycin (SMN,10 μg), erythromycin (ERY, 15 μg), ampicillin (AMP, 10 μg), gentamicin (GMN,10 μg), clindamycin (CMN, 2 μg), tetracycline (TET, 30 μg), trimethoprim (TMP, 5 μg), chloramphenicol (CHL, 30 μg), imipenem (IPM, 10 μg), and kanamycin (KMN, 30 μg) (Bio-Rad, CA, USA). After incubation at 37°C for 24 h, the inhibition zone was measured edge-to-edge across the antibiotic disks.

### *In vivo* administration in dogs

Based on *in vitro* results, the three strains were selected as potential probiotics for oral health in dogs. Ten healthy male dogs with poor oral conditions (plaque, tartar, and bad breath) and aged between 5 and 6 years were enrolled in this study. All dogs have never received oral care such as brushing or scaling during their lifetime. The health condition of the dogs was confirmed by a veterinarian based on the history of medication, surgery, and diarrhea. Bacterial strains were prepared once a week by culturing overnight in MRS broth at 37°C and washing twice with 0.85% NaCl. The resulting bacterial pellet was resuspended in 10% skim milk and 5% sucrose, and stored at −20°C in individual sterile tubes. Five dogs were randomly divided into a single (MJC175 only) or multi-strain groups (MJC175, MJC178, and MJC179). All dogs were orally administered 10^8^-10^9^ CFU of a single strain or multiple strains with water once a day for 4 weeks. Changes of oral pathogens were analyzed to evaluate the effects of the administrated probiotics on oral health. Their systemic effects were analyzed by comparing serum immunoglobulin and reproductive parameters before and after the administration. During the experiment period, the dogs were kept under daily observation (by the owner) for any abnormal symptoms caused by the administration of *L. acidophilus* isolates such as diarrhea, vomiting, and loss of appetite. In addition, on the last day of the experiment, the dogs were submitted to a general exam by a veterinarian. Two dogs included in the multi-strain group were excluded from the analysis because of regular deworming during the experiment. Dogs were routinely maintained with no changes in food or lifestyle before and after experiments. The owner's consent was obtained before the experiments were performed.

### Quantification of oral bacteria

Intraoral swab samples were collected before and after administering the single strain or multiple strains to analyze the changes in the dental caries pathogen (*S. mutans*) and three periodontal pathogens (*Actinomyces odontolyticus, Porphyromonas gingivalis*, and *Prevotella intermedia*). The same researcher swabbed the buccal sides of the right and left teeth using N-SWAB TRANSPORT (Noble Bio, Hwaseong, Korea) and stored the samples at −80°C until further analysis. DNA was extracted using the DNeasy Power Soil Kit (Qiagen, Hilden, Germany), following the manufacturer's protocol. Quantitative real-time PCR assays were performed according to previously reported species-specific primer pairs (31, 32): *S. mutans*: 5′-TCGCGAAAAAGATAAACAAACA-3′ and 5′-GCCCCTTCACAGTTGGTTAG-3′, *A. odontolyticus*: 5′-CTTTGGGATAACGCCGGGAAAC-3′ and 5′-CTACCCGTCAAAGCCTTGGT-3′, *P. intermedia*: 5′-CGTGGACCAAAGATTCATCGGTGG-3′ and 5′-ACCGCTTTACTCCCCAACAAA-3′, and *P. gingivalis*: 5′-AGGCAGCTTGCCATACTGCG-3′ and 5′-ACTGTTAGCAACTACCGATGT-3′.

### Measurement of serum IgG

To evaluate other potential *in vivo* effects of isolates, serum IgG levels were measured. In brief, blood was collected from the jugular vein before and after administering the single strain or multiple strains by a veterinarian and collected into a BD Vacutainer SST II Advance tube (BD, Plymouth, UK). Serum samples were separated by centrifugation, transferred to empty Eppendorf vials, and stored at −80°C until further experiments. Serum IgG was assayed using a Dog IgG ELISA Kit (Bethyl, Texas, USA), following the manufacturer's protocols.

### Evaluation of sperm quality

Additionally, sperm parameters were evaluated in an attempt a potential effect of isolates on the male reproductive function. Semen samples were collected and analyzed according to the method described by Mahiddine et al. (33). Briefly, the second fraction of semen was collected by a veterinarian before and after administering the single strain or multiple strains. After centrifugation and washing, the sperm were chilled in a Tris-egg yolk-glycerol extender and transported to the laboratory within 3 h. Sperm kinematic parameters, and sperm concentration were analyzed using Sperm Class Analyzer® System version 6.4.0.93 (Microptic, Barcelona, Spain).

### Statistical analysis

For statistical analysis, one-way analysis of variance (ANOVA) followed by Turkey's *post-hoc* test was used for antimicrobial activity results, acid, and bile salts tolerance results analysis. For serum IgG results and sperm analysis, a paired *t*-test was performed by comparing before and after isolates administration. Results were analyzed and visualized using GraphPad Prism 5 and R software version 4.1.2. All results were repeated at least three times and are presented as means and standard errors, and significant differences were determined using *P*-values (*p* < 0.05).

## Results

### General characterization of isolates

**Figure 1:**
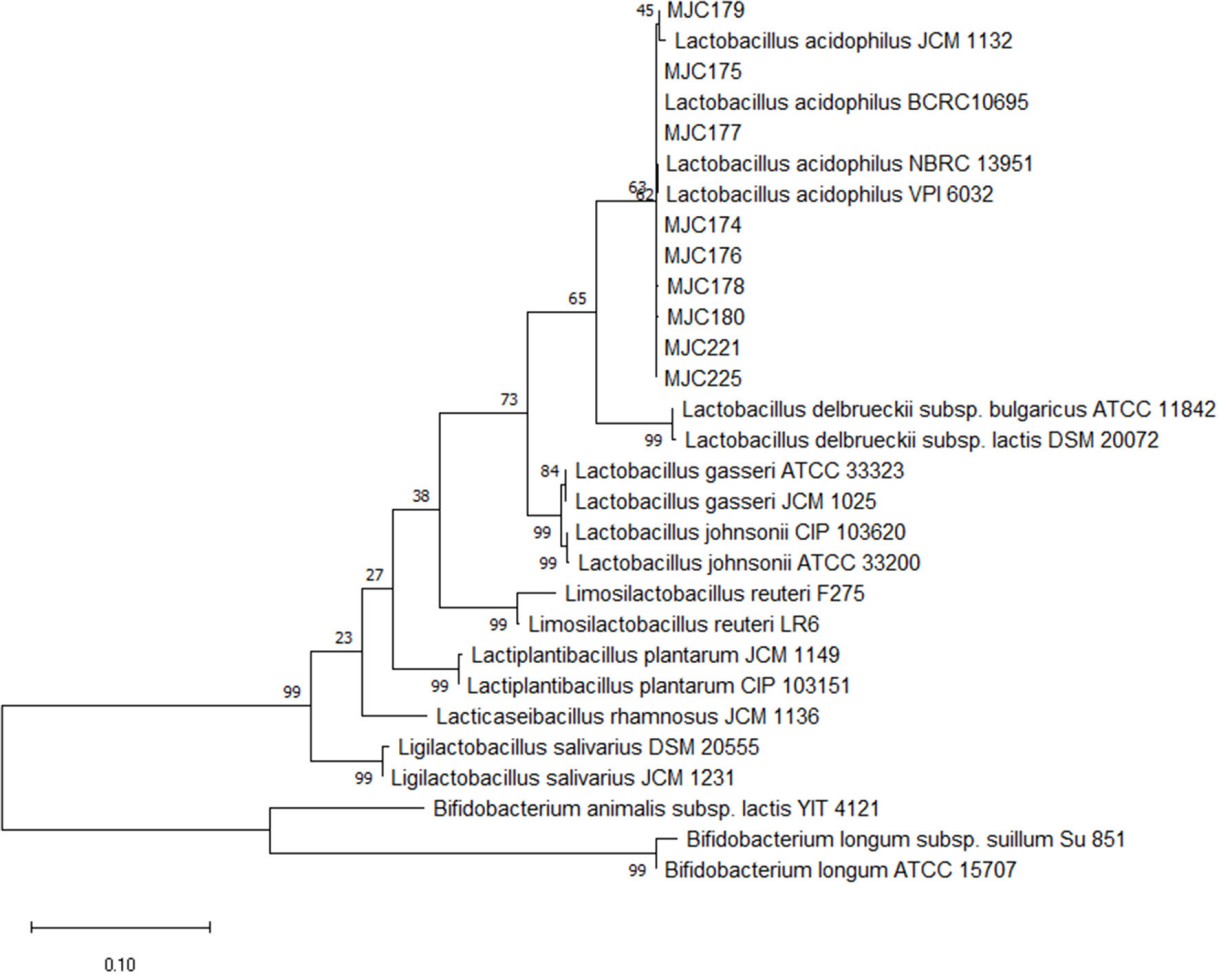
Phylogenetic tree of nine isolates. The 16S rRNA gene sequences of other genus and Lactobacillus species were downloaded from NCBI, and phylogenetic analysis with the maximum likelihood method *via* MEGAX software with a 1,000 bootstrap was performed.

Approximately 350 colonies were isolated from the feces of the 22 healthy dogs. Of these, 253 isolates were gram-positive cocci or bacilli-shaped bacteria. Except for strains that exhibited weak growth in MRS broth, 111 strains were subjected to PCR with *Lactobacillus* genus-specific primer pair. Based on the band size of the PCR products, 53 strains were presumed to be *Lactobacillus* and were used for molecular characterization using Sanger sequencing. Among the selected strains, nine were identified as *Lactobacillus acidophilus* with 99% sequence similarity and 100% coverage. A phylogenetic tree was constructed to demonstrate the relationship between the strains and other genus and species (Figure 1).

### Antimicrobial activity against four different pathogens

**Figure 2:**
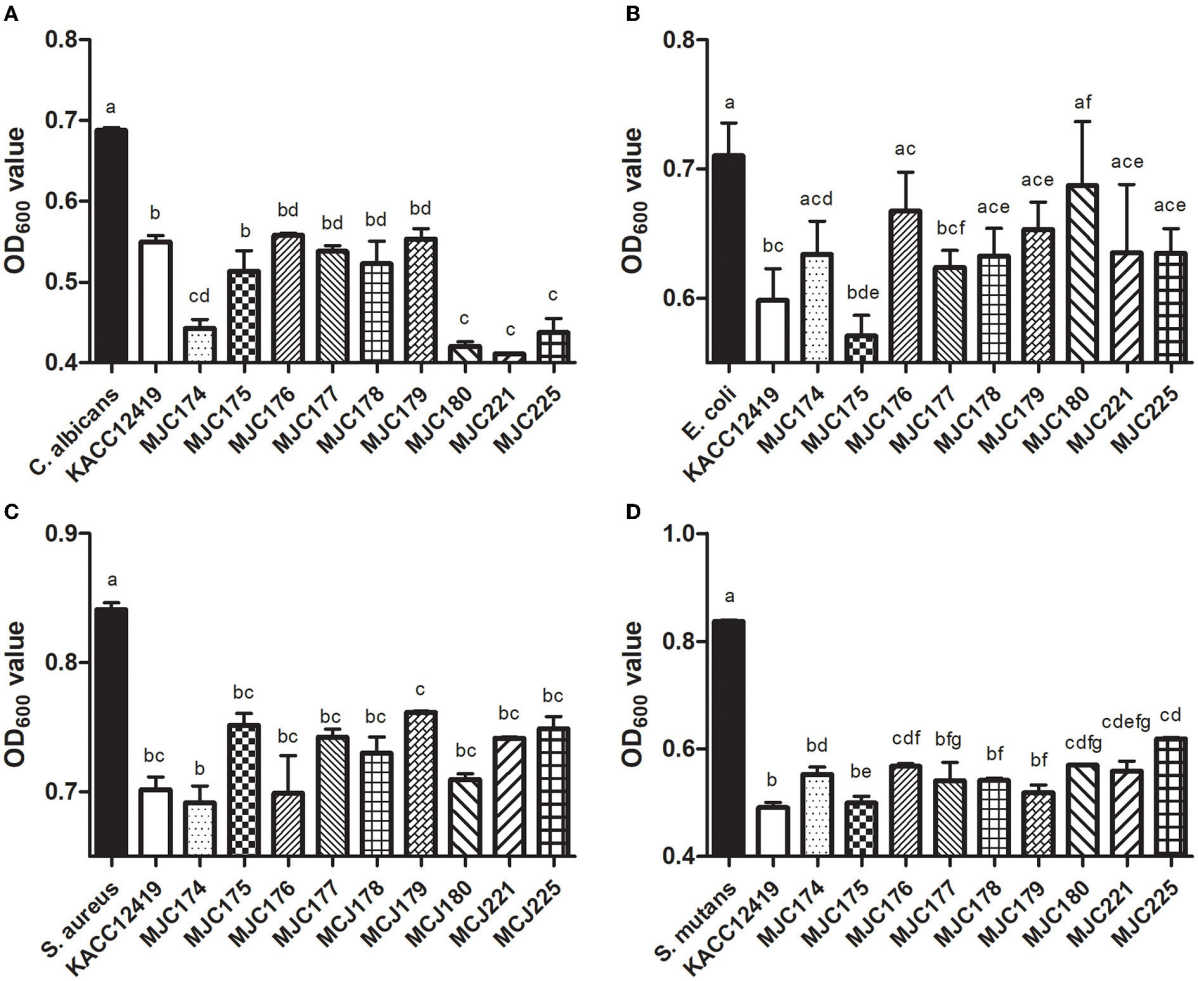
Antimicrobial activity of nine isolates against different pathogenic microorganisms. The supernatant of each isolate was used against **(A)** *Candida albicans*, **(B)** *Escherichia coli*, **(C)** *Staphylococcus aureus*, and **(D)** *Streptococcus mutans*. Lowercase letters indicate significant differences (*p* < 0.05) between strains. KACC; *L. acidophilus* KACC12419.

The inhibitory effect of supernatant of isolates against selected pathogenic microorganisms is shown in Figure 2. To determine the optimal inhibition time, pathogens in the supernatant were cultured for 18 and 24 h at 37°C, and the inhibition activity was higher at 24 h. After culturing for 24 h, all the isolates exhibited inhibitory activity against four different pathogenic microorganisms (*C. albicans, E. coli, S. aureus*, and *S. mutans*). Except for MJC180 in the inhibition of *E. coli*, all isolates significantly inhibited the four pathogenic microorganisms in a strain-dependent manner. In particular, these isolates strongly inhibited *S. mutans*, which is associated with the development of dental caries. *L. acidophilus* MJC175 possessed the highest inhibitory activity against *S. mutans*, followed by MJC179 and MJC178. *L. acidophilus* MJC175 also demonstrated more potent inhibition of *C. albicans* and *E. coli* than the *L. acidophilus* type strain (KACC 12419). Based on their antimicrobial activities against *S. mutans*, three isolates were selected and used in further experiments.

### Tolerance to gastrointestinal-like conditions

The tolerance results in low pH and bile salt conditions showed strain dependent (S1). In the pH 2 condition, MJC178 showed a lower survival rate (29.7%) than KACC 12419 (L. acidophilus KACC 12419, 54.7%), but the remaining two strains showed higher tendency (61.5% for MJC175 and 68.7% for MJC179). In the 0.3% of bile salt condition, all three strains showed similar suppression rate (30.9–45.9%) with no significant difference from KACC 12419 (33.4%). We assumed that three isolates can survive through the intestinal tract based on comparable tolerance with KACC 12419, and related analysis were analyzed after *in vivo* trial.

### Antibiotic susceptibility

The antibiotic resistance profiles of the selected isolates are presented in Table 1. The three isolates were selected based on their antimicrobial activity against *S. mutans*. All strains were sensitive to nine of 13 antibiotics. The isolates were most sensitive to AMP and IPM, and resistant to CIP, SMN, GMN, and KMN. The antibiotic sensitivity of the isolates was similar to *L. acidophilus* type stain (KACC 12419).

**Table 1:** Antibiotic resistance profiles of isolates toward 13 different antibiotics.

	**Diameter (mm) of inhibition zone**												
	**RIF**	**CIP**	**VAN**	**SMN**	**ERY**	**AMP**	**GMN**	**CMN**	**TET**	**TMP**	**CHL**	**IPM**	**KMN**
KACC 12419	14	R	24	R	24	34	R	10	30	17	25	38	R
MJC175	19	R	25	R	28	38	R	15	30	20	28	36	R
MJC178	22	R	23	R	31	42	R	14	32	28	32	40	R
MJC179	13	R	21	R	25	34	R	10	18	27	27	30	R

R; Resistant, KACC 12419; *L. acidophilus* KACC 12419.

### Effect of oral administration in dogs

**Figure 3:**
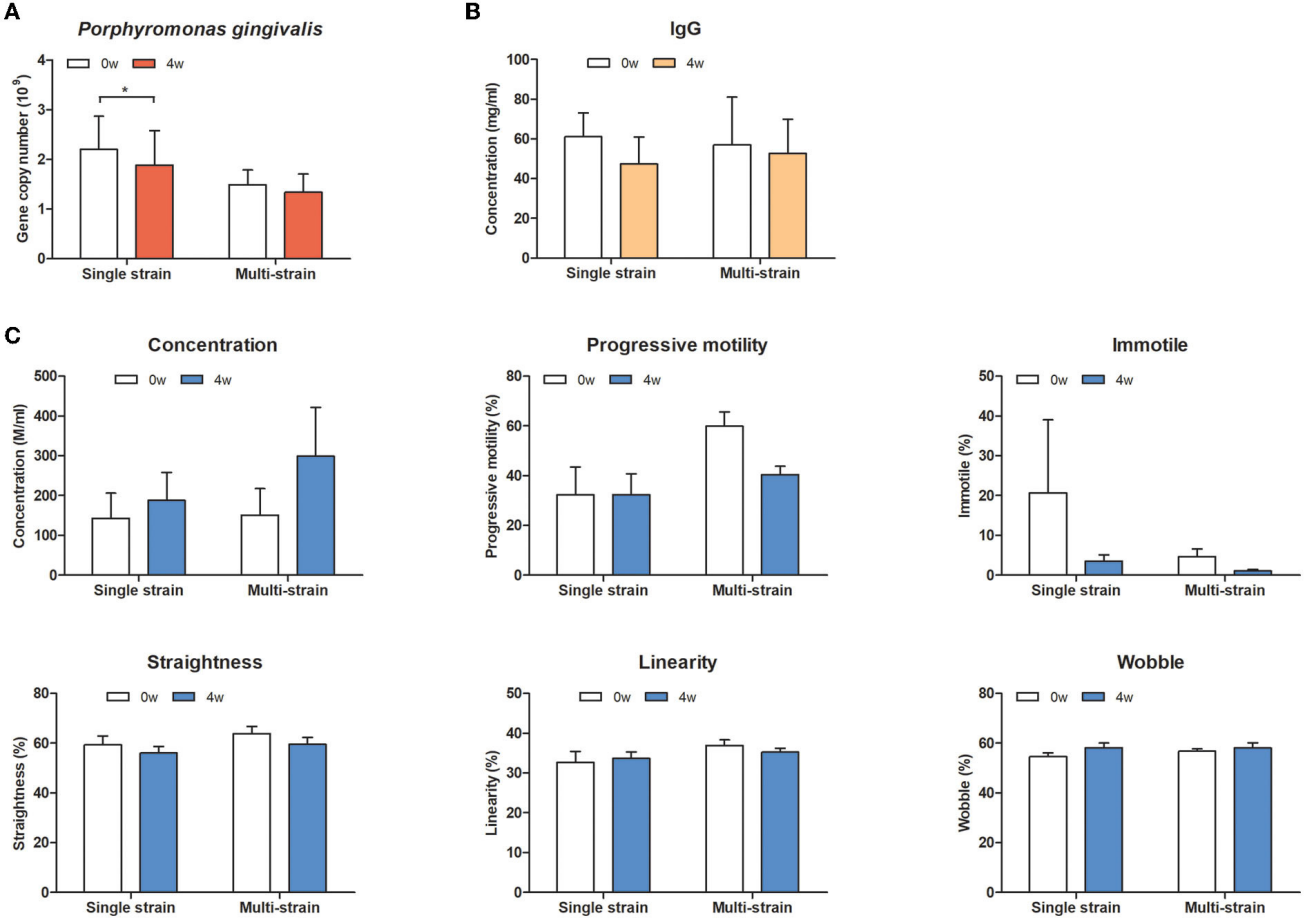
Effect of oral administration of a single strain or multiple strains in dogs. **(A)** The counts of *P. gingivalis*, **(B)** serum IgG level, and **(C)** sperm quality-relative parameters. Single strain; MJC175, Multiple strains; MJC175, MJC178, and MJC179.

To evaluate the effects of oral administration of the selected strains on oral health and systemic effects in dogs, oral bacteria, serum IgG levels, and sperm quality were analyzed. In the oral bacterial analysis, *S. mutans, A. odontolyticus*, and *P. intermedia* were not detected in any oral swab samples. However, DNA copies of *P. gingivalis* were significantly decreased in the single strain (MJC175) group at week 4 (Figure 3). There was no change in serum IgG levels in either group. There was a trend toward increased sperm concentration and decreased immotile spermatozoa at week 4 compared to week 0, but the difference was not statistically significant.

## Discussion

Next-generation sequencing technology provides a better understanding of host-microbe interactions and facilitates new therapeutic approaches (34). In recent decades, probiotics have been rationalized as a critical contributor to human and animal wellness. Many studies have been conducted to identify novel functional probiotic strains in response to market demand. Because the characteristics of each strain are different, even within the same species, selecting a strain that can produce the desired effects is essential (35, 36). Nevertheless, studies on probiotics for companion animals are limited compared to human studies. In the current study, we have evaluated the effectiveness of dog-derived strains as potential probiotics on the oral health of dogs, especially inhibition effects in *S. mutans* and *P. gingivalis* which are the leading pathogen associated with dental caries and periodontitis.

In the *in vitro* assay, the isolates strongly inhibited *S. mutans* among the four pathogenic microorganisms. Therefore, we assumed that *L. acidophilus* isolated from dogs had inhibitory activity against caries-causing bacteria and selected three strains with the highest inhibitory capacity. The supernatants of three isolates (*L. acidophilus* MJC175, *L. acidophilus* MJC178, and *L. acidophilus* MJC179) exhibited antimicrobial properties comparable to those of *L. acidophilus* type strain widely used as probiotics. Lactic acid bacteria produce functional compounds, such as functional proteins, polysaccharides, and microbial fractions during fermentation, which promote host health (37). In previous studies, the cell-free culture demonstrated antibacterial activity against oral pathogens and some metabolites, such as carbohydrates, fatty acids, and the level of hydrogen peroxide in the supernatant (38, 39).

*L. acidophilus* is considered safe and can be used as a feed additive in Korea (40). However, to ensure the safety of the selected strain, antibiotic susceptibility tests were performed prior to *in vivo* testing. All nine isolates possessed antibiotic resistance to CIP, SMN, GMN, and KMN. Most *Lactobacillus* species are intrinsically resistant to aminoglycosides (SMN, GMN, and KMN) and CIP (41). Aminoglycoside antibiotics cannot penetrate the membrane of lactobacilli, which may reduce their susceptibility (42). This is also considered to be an intrinsic resistance feature of *L. acidophilus* (36). In addition, the antibiotic resistance of bacterial strains has been documented as “intrinsic” or “natural,” according to the European Food Safety Authority (EFSA), strains with intrinsic resistance can be used as feed additives (43, 44).

Although candidate strains used in the *in vivo* trial were selected based on their antimicrobial activity against one of the strains, *S. mutans*, this strain was not detected in any participants. Interestingly, quantitative PCR revealed that administration of a single strain could reduce *P. gingivalis*, but there was no difference in the mix-strain group (Figure 2). The proportion of *P. gingivalis* increases in periodontal patients and this increase is proportional to the severity of the disease (45). Reduction of *P. gingivalis* may prevent the progression of periodontitis by inhibiting biofilm formation and dysbiosis induction of the oral microbiota. It is still unclear whether a single strain is more effective than a multi-strain mixture in general; however, in concordance with our results, some studies have reported that multiple strains are neither more effective nor significantly different from a single strain (46). Multi-strain probiotics can be mixed after the strains are cultured together or separately. Notably, the strains may have a competitive or synergistic relationship during the culture or after administration. For these reasons, a combination of several strains is not always better than a single strain, and trial evidence is essential for determining the appropriate probiotic.

The three selected isolates showed similar results in *in vitro* tolerance assays with *L. acidophilus* KACC 12419, one of the most used probiotic strains for gut health. Therefore, we predicted that these isolates could survive through the gastrointestinal tract, like KACC 12419, and that orally administered isolates would have the systemic benefits of probiotics. Systemic effects of probiotics were assessed by serum IgG and sperm quality assays, respectively. It is widely accepted that probiotics contribute to host immunity; a previous report showed that probiotic treatment improves immune response in dogs and leads to an increase in serum IgG levels (47). The term “gut-testicular axis”, which describes the relationship between the gut microbiota and the reproductive function, has been recently used in many studies. Additionally, we observed that oral administration of commensal beneficial bacteria improved sperm quality in male dogs in a previous study (48). The isolates are tolerant to harsh conditions, but even in the same species, they can have completely different effects on the host due to strain-dependent ability of the bacteria (35). As another possibility, the amount of administered isolate (10^8^-10^9^ CFU) may not be sufficient to affect those parameters. However, more studies are required to determine the effects of *L. acidophilus* MJCD175 on other benefits. These results suggest that these isolates are oral health-specific strains with no safety concerns in dogs.

In conclusion, the nine obtained *L. acidophilus* novel strains isolated from healthy dogs showed strong antimicrobial activity against caries-causing pathogens in an *in vitro* assay. Among them, *L. acidophilus* MJC175 inhibits high-risk periodontal pathogens when administered to dogs, thereby preventing periodontal disease that can be transmitted from dogs to dogs and humans. There were no adverse effects on immune response and reproductive function after administration to dogs. A commensal beneficial bacterium, *L. acidophilus* MJC175, can be considered specific and safe for dogs and can be used to compete with target pathogens. A novel potential probiotic strain, *L. acidophilus* MJC175, was renamed *L. acidophilus* MJCD175 based on encouraging results *in vitro* and *in vivo*. It can be applied as a host and disease-specific probiotic strain for oral health in dogs, making it an excellent choice for dental care in dogs that struggle to brush their teeth.

## Data availability statement

The raw data supporting the conclusions of this article will be made available by the authors, without undue reservation. The L. acidophilus MJCD175 strain is deposited in the Korean Agricultural Culture Collection, Wanju, Korea (accession No. KACC81211BP) for a patent. The genome sequence of L. acidophilus MJCD175 is deposited in NCBI GenBank under number ON556402.1.

## Ethics statement

Ethical review and approval of animal testing was not required as all animals enrolled in this study were privately owned dogs. Written consent was obtained from the owner for the animal's participation in this study.

## Author contributions

MK and IY: conceptualization, data curation, investigation, methodology, and visualization, sample collection, writing—review and editing, formal analysis, and validation. MK: supervision. IY: writing—original draft. FM: sample collection and methodology. MK and HP: funding acquisition, project administration, and resources. All authors contributed to the article and approved the submitted version.

## Funding

This study was supported by a cooperative research program of Rural Development Administration (#PJ015274022022).

## Conflict of interest

Authors IY, FM, HP, and MK were employed by Mjbiogen corp. All authors declare that the research was conducted in the absence of any commercial or financial relationships that could be construed as a potential conflict of interest.

## Publisher's note

All claims expressed in this article are solely those of the authors and do not necessarily represent those of their affiliated organizations, or those of the publisher, the editors and the reviewers. Any product that may be evaluated in this article, or claim that may be made by its manufacturer, is not guaranteed or endorsed by the publisher.
