# Supplementary material for: Lactobacillus acidophilus novel strain, MJCD175, as a potential probiotic for oral health in dogs
Source: Front Vet Sci. 2022 Sep 2;9:946890. doi: 10.3389/fvets.2022.946890 (PMC9478757; doi:10.3389/fvets.2022.946890)
Supplement: Supplementary file 1 [file Table_1.DOCX]

Supplementary Material

**
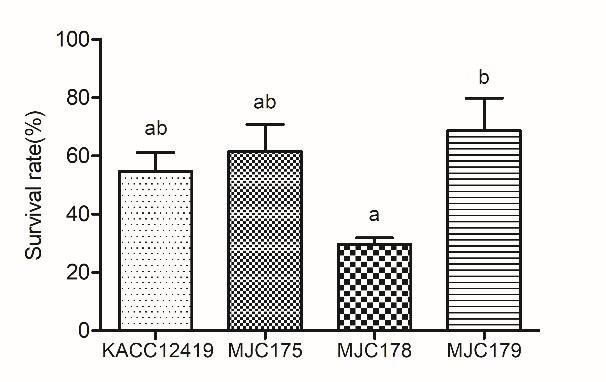

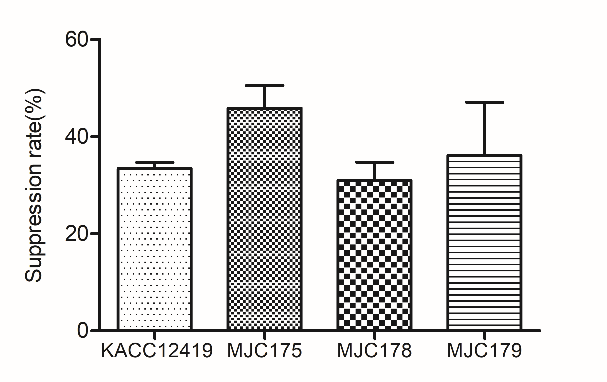
**

**Supplementary Figure 1****.** Tolerance to gastrointestinal-like conditions of three isolates. (a) Survival rate in low pH (pH 3) and (b) suppression rate in 0.3% bile salts. KACC12419; *Lactobacillus acidophilus* KACC12419.
